# Integrated Analysis of Cell Cycle–Related and Immunity-Related Biomarker Signatures to Improve the Prognosis Prediction of Lung Adenocarcinoma

**DOI:** 10.3389/fonc.2021.666826

**Published:** 2021-06-04

**Authors:** Fangyu Chen, Jiahang Song, Ziqi Ye, Bing Xu, Hongyan Cheng, Shu Zhang, Xinchen Sun

**Affiliations:** ^1^ Department of Radiation Oncology, The First Affiliated Hospital of Nanjing Medical University, Nanjing, China; ^2^ Department of Synthetic Internal Medicine, The First Affiliated Hospital of Nanjing Medical University, Nanjing, China; ^3^ Core Facility Center, The First Affiliated Hospital of Nanjing Medical University, Nanjing, China

**Keywords:** lung adenocarcinoma, cell cycle, immune infiltration, prognostic signature, bioinformatics

## Abstract

**Background:**

Lung adenocarcinoma (LUAD) is a leading malignancy and has a poor prognosis over the decades. LUAD is characterized by dysregulation of cell cycle. Immunotherapy has emerged as an ideal option for treating LUAD. Nevertheless, optimal biomarkers to predict outcomes of immunotherapy is still ill-defined and little is known about the interaction of cell cycle-related genes (CCRGs) and immunity-related genes (IRGs).

**Methods:**

We downloaded gene expression and clinical data from TCGA and GEO database. LASSO regression and Cox regression were used to construct a differentially expressed CCRGs and IRGs signature. We used Kaplan-Meier analysis to compare survival of LUAD patients. We constructed a nomogram to predict the survival and calibration curves were used to evaluate the accuracy.

**Results:**

A total of 61 differentially expressed CCRGs and IRGs were screened out. We constructed a new risk model based on 8 genes, including ACVR1B, BIRC5, NR2E1, INSR, TGFA, BMP7, CD28, NUDT6. Subgroup analysis revealed the risk model accurately predicted the overall survival in LUAD patients with different clinical features and was correlated with immune cells infiltration. A nomogram based on the risk model exhibited excellent performance in survival prediction of LUAD.

**Conclusions:**

The 8 gene survival signature and nomogram in our study are effective and have potential clinical application to predict prognosis of LUAD.

## Introduction

Lung cancer is a leading cause of cancer-related death over the world (1). It is reported that 2,206,771 lung cancer new cases and 1,796,144 deaths occurred in 2020 worldwide (2). Non-small cell lung cancer (NSCLC) is the most common histological type of lung malignancies, accounting for over 80% of all cases, and near half of NSCLC are lung adenocarcinoma (LUAD) (3, 4). The prognosis of LUAD has been disappointing over the past two decades, with a five-year survival rate below 20% (5). Mortality from LUAD has decreased substantially in recent years, coinciding with the advances in immunotherapy. Immune checkpoint inhibitors (ICIs), especially inhibitors of the programmed cell death 1 (PD-1) axis have altered the therapeutic landscape of LUAD (6, 7). The pacific clinical trial reported that PD-1 inhibitor pembrolizumab improves three-year overall survival of NSCLC from 43.5% to 57.0% (8). However, only a subset of patients achieves an impressive and durable response to immunotherapy (9).

A major molecular characteristic of human cancer is that key cell cycle proteins are frequently dysregulated. Dysfunction of the cell cycle regulators forces tumor cells enter uncontrolled cell division (10, 11). The mitotic stress and chromosomal instability result in replication errors and increased mutation load (12). Tumor mutation load and tumor-infiltrating lymphocytes (TILs) have been widely investigated as prognostic and predictive biomarkers in multiple tumor types, including lung cancer (13, 14). In addition, cell cycle-targeted therapy such as cyclin-dependent kinase (CDK) 4 or CDK6 inhibitors induce tumor cell novel-antigen and recruit TILs, indicating a possible option for immunotherapy combination. Previous studies have suggested that the cell cycle regulator RB might also be required for tumor cells’ expression of MHC Class II molecules, which can be recognized by TILs and mediate cytotoxic killing of tumor cells. Additionally, the increased tumor neoantigen observed after CDK4/6 blockade increases the efficacy of ICB. One report has reported that CDK4/6 inhibition potentiates the expression of PD-L1 in tumor cell together with a decrease of T cell function in the tumor microenvironment (15–18). Given the critical association of cell cycle regulation and tumor immunotherapy, it is highly desirable and urgent to exploit cell cycle regulators and TILs in LUAD. However, the comprehensive analysis of cell cycle-related genes (CCRGs) and immune-related genes (IRGs) or the interactions between them are still not known. In the current research, we comprehensively analyzed the CCRGs and IRGs in LUAD. In addition, we established a predictive signature based on them and further depicted the potential regulatory network between IRGs and specific CCRGs in LUAD. This work provided novel insights into predicting the prognosis and efficacy of immunotherapy in LUAD patients, which brings up a new prospect for enhancing the personalized medication for the treatment of LUAD.

## Methods

### Data Extraction and Processing

The RNA-seq profiles and related clinical characteristics of LUAD patients were extracted from TCGA and GSE68465. The IRGs for further immunological analysis were based on the ImmPort database (https://www.immport.org/home) (19). The reference CCRGs set were retrieved from MSigDB2. “GO_CELL_CYCLE” was also picked from “all GO gene sets as Gene Symbols” in “c5: Ontology gene sets. The | log2 fold change | > 0.5, *p* value < 0.05 and false discovery rate (FDR) <0.05 were defined as the cut-off vales to screen out the differentially expressed genes (DEGs) of LUAD. Differentially expressed CCRGs and IRGs were defined as the intersection of DEGs, CCRGs and IRGs. Gene ontology (GO) and Kyoto Encyclopedia of Genes and Genomes (KEGG) were used to identify the enriched function of these genes by using Enrichr database (http://amp.pharm.mssm.edu/Enrichr/) (20–22).

### Identification of Interaction Network of DEGs

The STRING database (https://string-db.org/) was obtained to investigate protein-protein interactions (PPIs) based on the intersection of DEGs, CCRGs and IRGs (23). In the present study, an PPI-score greater than 0.9 was set as the threshold. The Cytoscape software was used to distinguish the hub genes and visualize the PPI results (24).

### Construction and Verification of the CCRGs and IRGs Prognostic Signature

The data of LUAD cases were downloaded from GEO (GSE68465, n=439) and TCGA (n=504). The GEO database was used as a train set and the TCGA database were used as a test set. LASSO Cox regression and multivariate Cox analysis were used to identify hub genes to construct the predictive signature. First, we used univariate Cox proportional hazard regression to identify prognosis-related genes with the cut-off value of *p* < 0.05. To eliminate the overfit gene of the model, these prognosis-related genes from univariate Cox analysis were further included in LASSO penalized Cox proportional hazards regression *via* R package “glmnet”. Next, the optimal model based on the remained genes from LASSO analysis was constructed by multivariate Cox regression analysis backward stepwise regression. The formula for risk score is: $\text{risk}\;\text{score}=\Sigma_{i=1}^{\text{N}}\;Exp_{i}\times Coe_{i},N,Exp_{i},\;\text{and}\;Coe_{i}$ represents optimal gene numbers, expression levels of gene, and regression coefficients, respectively. Meanwhile, patients were separated into low- and high-risk groups according to the average value. We employed Kaplan-Meier analysis to weigh the differences between survival of each group, and ROC curve of 1-year, 3-year and 5-year survival was performed to assess the efficacy. In addition, we did univariate and multivariate Cox regression to analyze the risk score of prognostic models and several clinicopathological characteristics for LUAD. The risk model based on train group was validated in the test group and entire group. We used the R packages “rms” to construct a nomogram to predict prognosis of LUAD patients. A calibration map was generated by comparing the nomogram predictive efficacy for the 1-year, 3-year and 5-year OS rates (25).

### Infiltrating Immune Cells Signature Analysis

The data of infiltrating immune cells in the tumor microenvironment of LUAD patients were downloaded from the TIMER database (https://cistrome.shinyapps.io/timer) (26). We employed Spearman’s test to evaluate correlations between genes and the infiltrating immune cells. A two-tailed *p* value lower than 0.05 was set as the cut-off value for statistical significance.

### Tumor Mutation Burden Analysis

The tumor mutation profile of LUAD patients were obtained from the TCGA database. The somatic mutations of LUAD were analyzed from mutation annotation format. We used the formula: tumor mutation burden $(TMB)=\frac{total\;mutation}{total\;coveredbases}\times 10^{6}$ to determine tumor mutation TMB for each LUAD case.

## Results

### Identification of Differentially Expressed Cell Cycle-Related and Immunity-Related Genes

**Figure 1:**
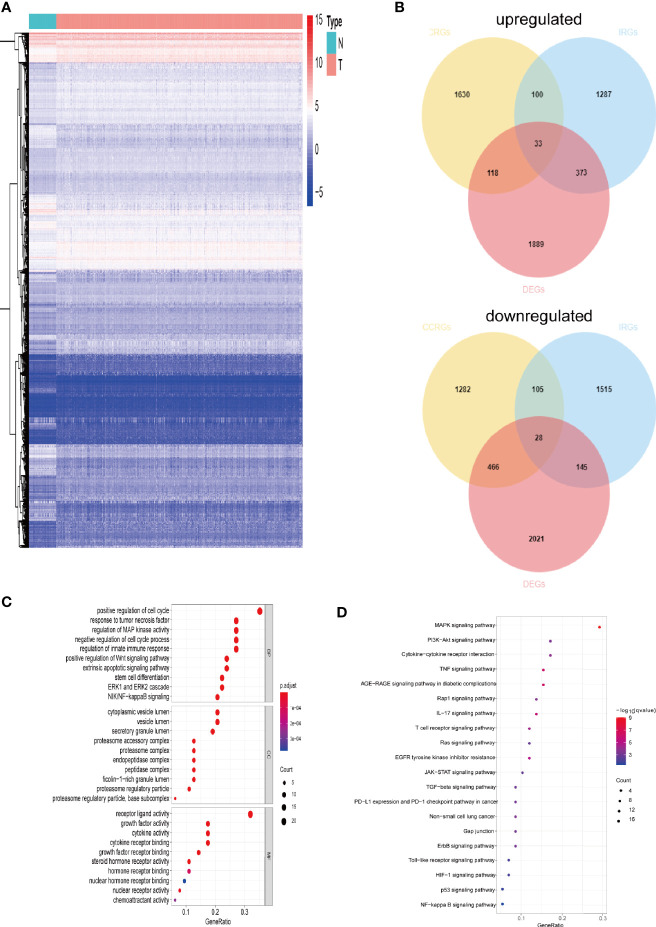
Comparison of gene expression profile with CCRGs and IRGs of LUAD. **(A)** Heatmap of differentially expressed genes in LUAD. **(B)** Venn diagram of up- and down-regulated differentially expressed genes based on CCRGs and IRGs. **(C)** GO analysis. **(D)** KEGG analysis.

**Figure 2:**
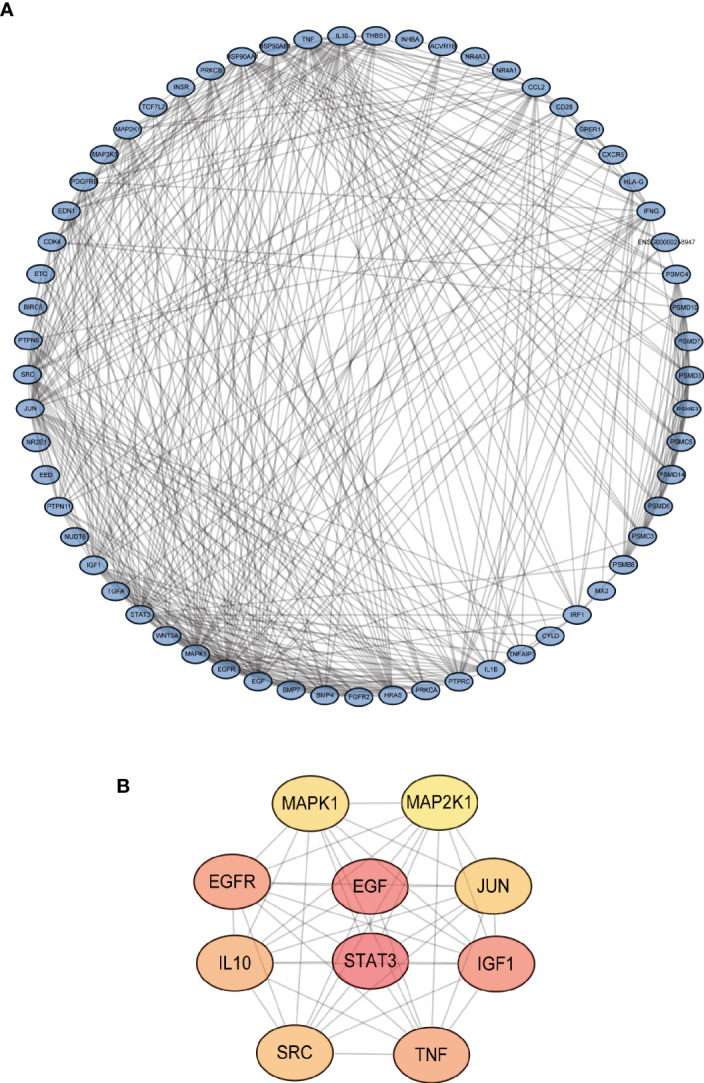
Identification of PPI network of the DEGs. **(A)** Visualization of the PPI network using STRING database and Cytoscape software. **(B)** Interactions of the top ten hub genes.

The gene expression levels of LUAD (439 samples) and normal lung tissues (19 samples) in GEO database (GSE68465) were analyzed. The results demonstrated 3,910 DEGs, 1,889 of which are upregulated and 2,021 are downregulated (**Figure 1A**). Then, the intersection with two sets of CCRGs and IRGs revealed 33 upregulated and 28 downregulated DEGs that are involved in regulation of cell cycle and immunity (**Figure 1B**). GO analysis demonstrated that the differentially expressed CCRGs and IRGs are mainly enriched in **Figure 1C**. The significant KEGG pathways were enriched in **Figure 1D**. To further explore the interactions among the 61 differentially expressed CCRGs and IRGs, we established the PPI network (**Figure 2A**). Ten hub genes (EGF, EGFR, STAT3, IGF1, TNF, IL10, SRC, JUN, MAPK1, MAP2K1) were identified using the Cytoscape software (**Figure 2B**).

### Identification and Evaluation of the CCRGs and IRGs Prognostic Signature

**Figure 3:**
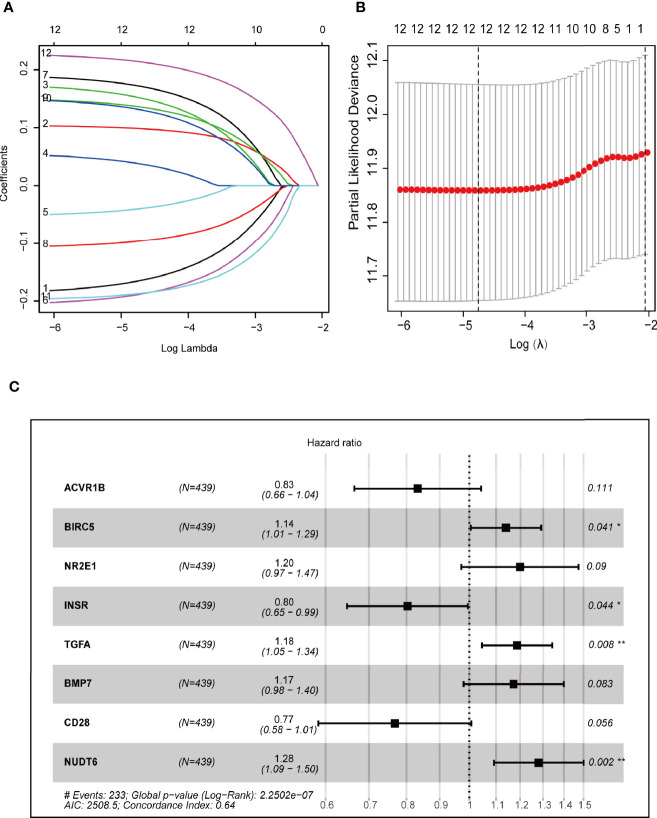
Construction of prognostic models based on CCRGs and IRGs. **(A, B)** LASSO Cox regression analysis based on OS. **(C)** Forest plots presenting the multivariate Cox proportional hazards regression analysis of prognostic CCRGs and IRGs in OS.

**Figure 4:**
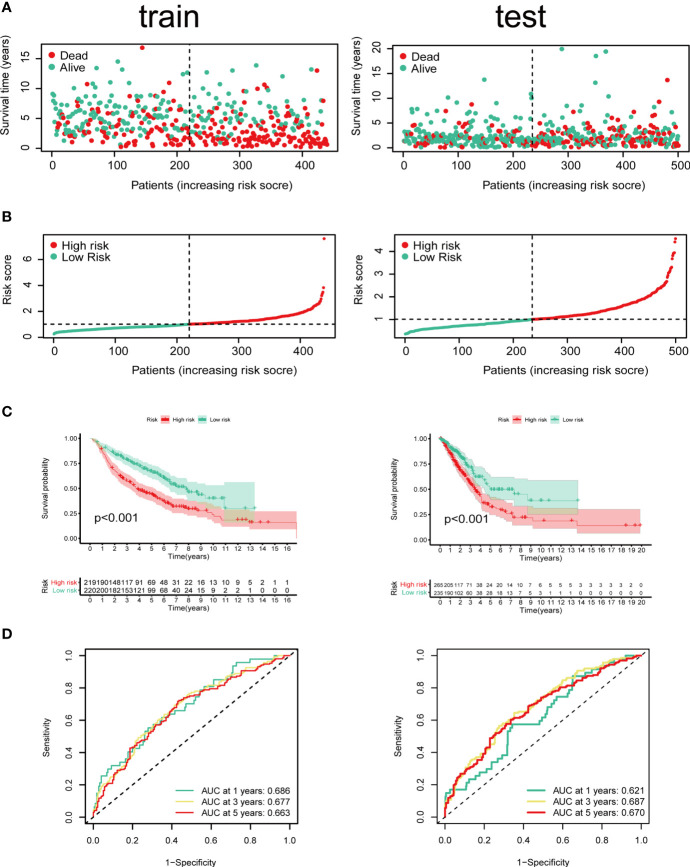
Identification of prognostic model in train set and test set based on OS of LUAD in GEO and TCGA cohorts. **(A)** The survival status and survival time of patients with LUAD ranked by risk score. **(B)** Rank of risk score and distribution of groups. Patients with LUAD were divided into high- and low-risk groups based on the median value of the risk score calculated. **(C)** Kaplan-Meier analysis and **(D)** time-dependent ROC curve of risk score.

Cox regression and LASSO regression were performed in the train set to establish a CCRGs and IRGs predictive signature based on the 61 DEGs to evaluate the survival of LUAD patients. First, we used univariate Cox proportional hazard regression to identify prognosis-related genes from 61 DEGs. With the cut-off value of *p* < 0.05, the 12 prognosis-related genes were identified (ACVR1B, BIRC5, NR2E1, PTPN11, STAT3, INSR, MAP2K1, PRKCB, TGFA, BMP7, CD28 and NUDT6). To eliminate the overfit gene of the model, these 12 prognosis-related genes from univariate Cox analysis were further included in LASSO penalized Cox proportional hazards regression *via* R package “glmnet” (**Figures 3A, B**). Next, the optimal model based on the remained genes from LASSO analysis (ACVR1B, BIRC5, NR2E1, PTPN11, STAT3, INSR, MAP2K1, PRKCB, TGFA, BMP7, CD28 and NUDT6) was constructed by multivariate Cox regression analysis backward stepwise regression. Consequently, eight genes (ACVR1B, BIRC5, NR2E1, INSR, TGFA, BMP7, CD28, NUDT6) were identified for the risk model (**Figure 3C**). The risk score is = (-0.1831 × ACVR1B expression) + (0.1300 × BIRC5 expression) + (0.1798 × NR2E1 expression) + (-0.2200 × INSR expression) + (0.1697 × TGFA expression) + (0.1577 × BMP7 expression) + (-0.2649 × CD28 expression) + (0.2473 × NUDT6 expression). Patients were separated into high- and low-risk groups based on the average risk score. The survival of each group was shown in **Figures 4A, B**. The Kaplan-Meier survival analysis and the log-rank test demonstrated that patients in the low-risk group had significantly longer survival time (p<0.05, **Figure 4C**). In addition, the 1-, 3-, and 5-year ROC curves were plotted as **Figure 4D**. The test set proved the risk model based on the train set.

### Subgroup Analysis of the Prognostic Signature

**Figure 5:**
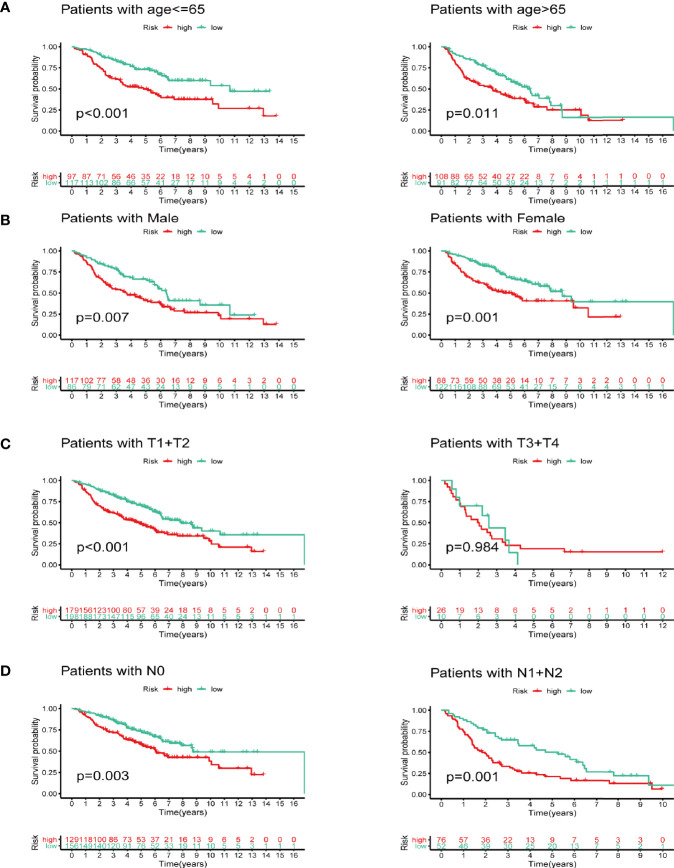
Subgroup survival analysis based on different clinicopathological features of LUAD. **(A)** Age. **(B)** Gender. **(C)** T stage. **(D)** N stage.

We next did subgroup survival analysis based on different clinical features using the signature. These subgroups included age (≤65 or >65), gender, T stage (T1-2 or T3-4), and N stage (N0 or N1-2). As shown in **Figure 5**, the 5-year survival of the high-risk group stratified by the subgroups mentioned above were significantly shorter than that of the low-risk group. However, due to the small number of samples in the T3-4 group (n=36), the survival between two groups did not show significant differences.

### Construction and Evaluation of the Prognostic Nomogram

**Figure 6:**
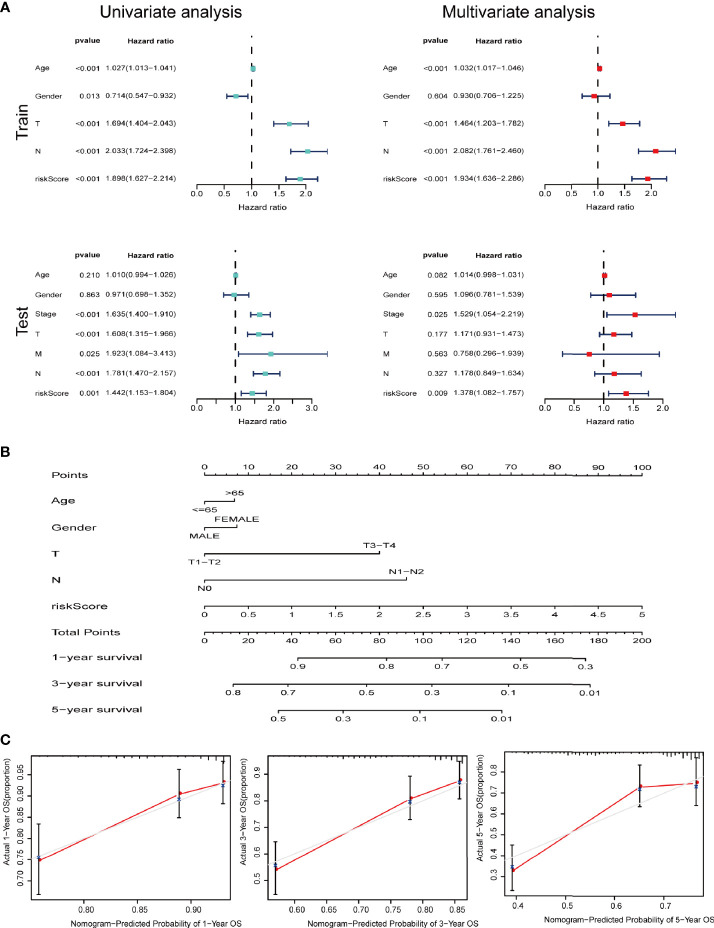
IRGs and CCRGs combined with other clinical factors to predict prognosis of LUAD patients based on OS model. **(A)** Univariate and multivariate Cox regression revealed significant survival-related clinicopathological parameters in forest plots diagram. **(B)** Nomogram and **(C)** calibration curve was constructed to verify the accuracy of predicting 1-, 3-, and 5-year survival rates.

We further performed univariate and multivariate Cox analysis to investigate whether signature is an independent risk factor for the survival in LUAD. Univariate Cox analysis indicated that age, T stage, N stage and the signature were meaningful for predicting OS (p<0.001). Multivariate Cox analysis identified that the signature was an independent risk factor for predicting survival of LUAD (**Figure 6A**). As shown in **Figure 6B**, we used the clinicopathological features and risk score to generate a nomogram to predict the survival of LUAD patients. The worse prognosis is associated with higher sample scores. A calibration curve was used to evaluate the efficacy of the predictive model. It demonstrated that the signature in our study is better in predicting the 1-year, 3-year, and 5-year survival (**Figure 6C**).

### The Infiltrating Immune Cells Signature and Tumor Mutation Burden Profile

**Figure 7:**
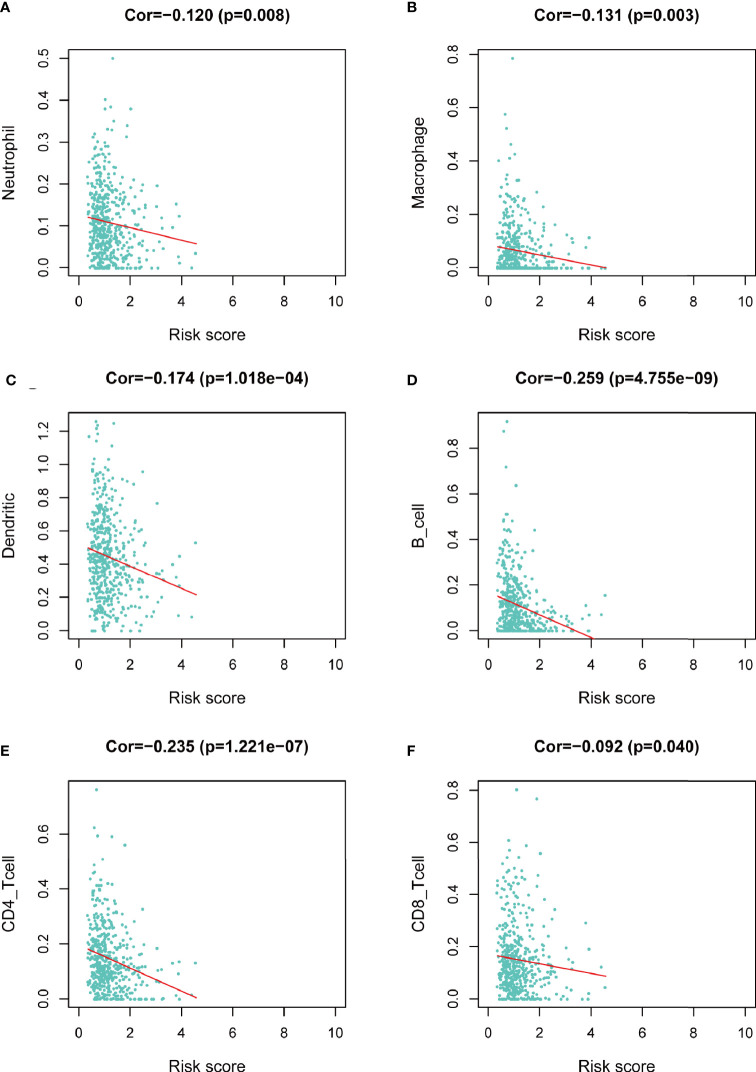
Correlation between risk score and infiltrating immune cells in the LUAD tumor microenvironment. **(A)** Neutrophil. **(B)** Macrophage. **(C)** Dendritic cell. **(D)** B lymphocyte. **(E)** CD4^+^ T lymphocyte. **(F)** CD8^+^ T lymphocyte.

**Figure 8:**
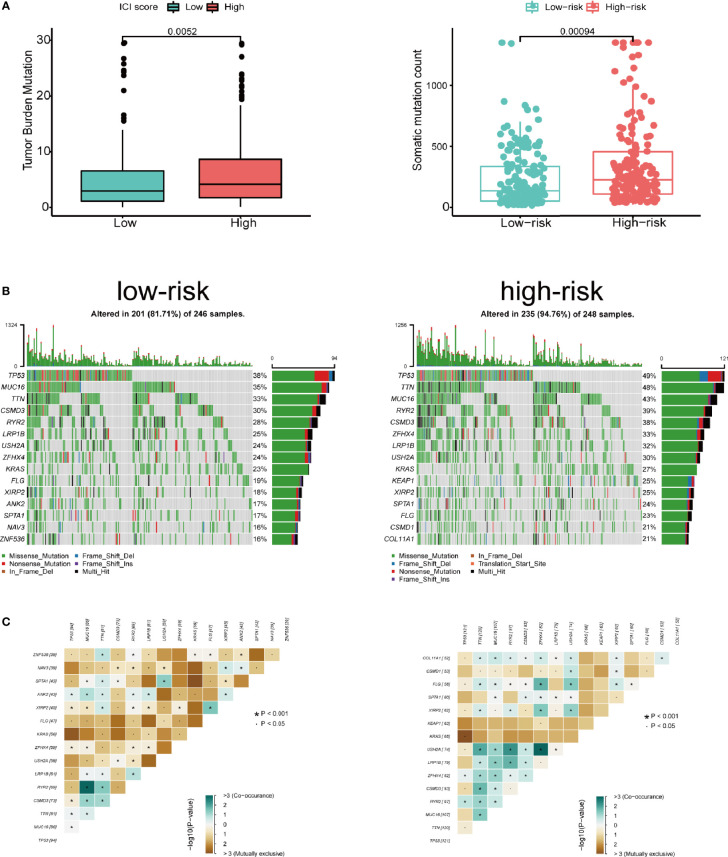
Tumor mutation in the high- and low-risk groups. **(A)** Patients in high-risk group showed significantly higher tumor mutation burden and somatic mutation count. **(B)** TP53, MUC16, TTN, RYR2, and CSMD3 ranked the top five TMB genes in both groups, and the TMB of them was drastically higher in high-risk group. **(C)** Correlations between mutated genes in both groups.

We assessed the correlation between risk score and infiltrating immune cells in the tumor microenvironment using TIMER database. We found that patients with high-risk score was negatively correlated with infiltrating neutrophils, macrophages, dendritic cells, B lymphocytes, CD4^+^ T lymphocytes and CD8^+^ T lymphocytes in the tumor microenvironment (p<0.05), indicating a universal decrease of infiltrating immune cells (**Figures 7A–F**). Next, we further investigated the TMB of differentially expressed CCRGs and IRGs based on the risk signature. Immune checkpoint inhibitors have demonstrated significant overall survival benefit in LUAD. Nevertheless, a remarkable interpatient heterogeneity characterizes immunotherapy efficacy. TMB is an essential factor related to outcomes of immunotherapy of lung cancer. Since LUAD mostly occurs in non-smokers, the TMB of LUAD is relatively lower than that of lung squamous cell carcinoma, highlighting the necessity to explore factors related to TMB. We demonstrated that TMB and somatic mutation count was remarkably higher in the high-risk group (**Figure 8A**). TMB of genes in both groups are listed in **Figure 8B**. TP53, MUC16, TTN, RYR2, and CSMD3 ranked the top five TMB genes in both groups, and the TMB of them was drastically higher in high-risk group. Then, the correlations between TMB of genes were assessed. As shown in **Figure 8C**, TP53 and KRAS showed the most positive correlation, and RYR2 exhibited negative correlation with MUC16.

## Discussion

Lung cancer is characterized as one cancer type which has high morbidity and mortality. Almost 85% of lung cancer subtypes are NSCLC, of which LUAD and lung squamous cell carcinoma are the most common subtypes (3). LUAD is the most common histological subtype in never-smokers. With the progress of anti-smoking campaign, the incidence of LUAD is rapidly climbing. Despite advances in cancer treatment, the prognosis of NSCLC has been disappointing over the past decades: the 5-year survival rate for patients with metastatic LUAD was less than 5% (27). Immunotherapy has changed the landscape for treating advanced NSCLC. The application of ICBs such as antibodies against PD-1 or PD-L1 have pointed a new direction for LUAD care. Nevertheless, the response to ICBs varies among LUAD patients. Novel biomarkers to predict the outcomes of immunotherapy is urgently needed. Cell cycle dysfunction is tightly associated with tumorigenesis in lung cancer. Loss of RB protein is found in approximately 15%-20% of all NSCLC (28). The dysregulation of cell cycle drives cancer cells into uncontrolled proliferation. The rapid division and genome instability of tumor cells result in mutation of DNA mutation. TMB is reported to be a promising immunotherapy biomarker to predict survival across multiple cancer types. In addition, cell cycle-targeting drugs CDK4/6 inhibitor may enhance the expression of PD-L1 on tumor cells, indicating the significance of combining ICBs. However, the interactions between cell cycle and immune regulators are still ill-defined.

In this study, we analyzed the differentially expressed CCRGs and IRGs in LUAD of GEO and TCGA cohorts. We identified 28 upregulated and 33 downregulated differentially expressed CCRGs and IRGs. GO and KEGG functional enrichment analyses revealed potential mechanism of them in LUAD. MAPK signaling pathway is mostly involved in these DEGs. Stutvoet et al. reported that MAPK pathway plays a significant role in PD-L1 expression of LUAD and may become a target to improve the outcomes of immunotherapy (29). We subsequently did a PPI network analysis among the 61 differentially expressed CCRGs and IRGs and 10 hub genes stand out (EGF, EGFR, STAT3, IGF1, TNF, IL10, SRC, JUN, MAPK1, MAP2K1). EGF and EGFR are the most important mutations in NSCLC, especially LUAD. Target therapy against EGFR mutation have greatly improved LUAD survival (30). STAT3 can induce epithelial mesenchymal transition and participate in PD-1 signaling pathway (31).

We then generated a risk model based on 61 differentially expressed CCRGs and IRGs based on GEO cohorts, and TCGA cohorts were obtained to verify the survival predictive value of the risk model. The train set revealed eight key genes (ACVR1B, BIRC5, NR2E1, INSR, TGFA, BMP7, CD28, NUDT6). ACVR1B, also known as ALK4, encodes an activin receptor related in TGF-β superfamily or structural related signaling proteins. Mutations in this gene are associated in with progressive pancreatic cancer in mutant KRAS-induced patients (32). Similarly, a SNP variant was found in never-smoking lung cancer patients, indicating the potential driving role of ACVR1B in NSCLC (33). BIRC5, also known as survivin, is a member of the inhibitor of apoptosis gene family that prevents apoptosis and has functions in both cell survival and mitosis. BIRC5 is normally absent in mature cells but is distinctly overexpressed in tumor cells. A small-molecule inhibitor of BIRC5, YM155, was shown to have favorable safety/tolerability in NSCLC patients (34). In addition, YM-155 had radio-sensitizing effect in NSCLC cell lines (35). NR2E1 and INSR are both proliferation regulators involved in aggressive behaviors (36, 37). TGFA is a ligand for EGFR. Dopeso reported that EMT-induced upregulation of TGFA can stimulate EGFR, activate SMAD pathway and induce EMT, which forces a positive feedback loop to enhance EMT and metastasis of lung cancer (38). BMP7 encodes a ligand of TGF-beta superfamily and activate SMAD signaling pathway. CD28 is a famous protein required for T cell proliferation and mature, cytokine secretion and Th2 cell differentiation. Kamphorst showed that CD28 pathway is significant for effective PD-1 therapy. They reported that CD28 is required for the proliferation of CD8^+^ T cells and the increased T cells after PD-1 blockade therapy are mainly CD28 positive (39). NUDT6 is an antisense gene of fibroblast growth factor 2 (FGF2). It is reported that NUDT6 is abundantly expressed in esophageal adenocarcinoma and is associated with poor disease-free survival (40). Taken together, the eight key genes are mainly involved in TGF-β pathway. The TME is characteristically enriched by TGF-β that are secreted by cancer cells, fibroblasts, macrophages and platelets. TGF-β can inhibit the differentiation of Th1 cells and cytotoxic CD8^+^ T cells and block T cell proliferation. Thus, it functions as a critical suppressor in the immune system and promotes immune evasion during cancer development. However, TGF-β has a contrary role as a tumor suppressor by inducing cell cycle arrest in early cancer cells (41, 42). The complexed role of TGF-β makes it significant to clarify its function in the future.

We next did a Kaplan-Meier analysis and it indicated that the survival of patients in low-risk group is remarkably longer than that in high-risk group. Additionally, the ROC curve and AUC verified accuracy of the risk model in survival prediction. We next constructed subgroup analysis based on the eight-gene signature. The high-risk group was also related to disappointing prognosis of LUAD patients with different age, gender, T stage and N stage. In addition, the model was proved to be an independent factor for OS. However, due to the small number of enrolled patients among T3-4 stage subgroup in the dataset (n=36), we did not find any significant difference, but the signature still worked for T1-2 subgroup LUAD patients. We further established a nomogram to determine a score for predicting survival of LUAD. The calibration curve implied the signature based on the risk model achieved a promising fit and greater effectiveness in clinical applications. In addition, we obtained TIMER database to study the correlation between the signature and infiltrating immune cells. We found that the infiltrating immune cells in the tumor microenvironment of LUAD is negatively associated with the risk score. To further explore the efficacy of our signature in immunotherapy, we performed a tumor mutation analysis. We found that LUAD patients in the high-risk group exhibited significantly higher TMB and more somatic mutation count. Since the infiltrating immune cells are main effectors in cancer immunotherapy and TMB is currently considered as an effective outcome index for immunotherapy, the signature in the current research might be useful in predicting the outcomes of LUAD patients receiving ICB medication.

Although the signature seemed to be stable and practical in our study, there are some limitations. The samples in our study were collected from TCGA databases and were internally verified. Further external verifications are needed to evaluate the accuracy in other databases. And the mechanisms and interactions of the eight key genes for constructing the risk models are needed to be elucidated by future experiments.

To conclude, we constructed a signature based on eight differentially expressed CCRGs and IRGs to predict the survival of LUAD. The risk models in our study show better clinical practicability for predicting the outcomes of LUAD patients. This signature might bring about changes in personalized LUAD treatment.

## Data Availability Statement

The datasets presented in this study can be found in online repositories. The names of the repository/repositories and accession number(s) can be found in the article/supplementary material.

## Author Contributions

FC, JS, HC, SZ, and XS contributed to the design of the study. JS and ZY extracted the data profile. FC, JS, and BX performed the statistical analysis. FC, JS, and XS contributed to the writing and revision of the manuscript. All authors contributed to the article and approved the submitted version.

## Funding

This work was supported by the National Natural Science Foundation of China (82073344).

## Conflict of Interest

The authors declare that the research was conducted in the absence of any commercial or financial relationships that could be construed as a potential conflict of interest.
